# High expression of Fatty Acid‐Binding Protein 5 promotes cell growth and metastatic potential of colorectal cancer cells

**DOI:** 10.1002/2211-5463.12031

**Published:** 2016-02-11

**Authors:** Koichiro Kawaguchi, Shogo Senga, Chiaki Kubota, Yuki Kawamura, Youqiang Ke, Hiroshi Fujii

**Affiliations:** ^1^ Interdisciplinary Graduate School of Science and TechnologyShinshu UniversityMinami‐minowaKami‐inaNaganoJapan; ^2^ Department of Bioscience and BiotechnologyFaculty of AgricultureShinshu UniversityMinami‐minowaKami‐inaNaganoJapan; ^3^ Molecular Pathology LaboratoryDepartment of Molecular and Clinical Cancer MedicineThe University of LiverpoolUK; ^4^ Department of Interdisciplinary Genome Sciences and Cell MetabolismInstitute for Biomedical SciencesInterdisciplinary Cluster for Cutting‐Edge ResearchShinshu UniversityMinami‐minowaKami‐inaNaganoJapan

**Keywords:** colorectal cancer, FABP5, fatty acid, fatty acid‐binding proteins, lipid metabolism, metastasis

## Abstract

Fatty acid‐binding proteins (FABPs) are responsible for binding and storing hydrophobic ligands such as long‐chain fatty acids, and for transporting these ligands to the appropriate compartments within the cell. The present study demonstrates that the *FABP5* gene is upregulated in colorectal cancer cells compared to normal colon cells in a manner that correlates with disease stage and that FABP5 significantly promotes colorectal cancer cell growth and metastatic potential. Thus, *FABP5* might be a promising prognostic or therapeutic biomarker candidate in human colorectal cancer.

**Abbreviations**
CRC: colorectal cancer
FABPs: fatty acid‐binding proteins
IBABP (FABP6): ileal bile acid‐binding protein

Colorectal cancer (CRC) is the third most frequent malignancy and fourth leading cause of cancer‐related death worldwide 1. Genetic mutations, epigenetic alterations, inflammation, and environmental factors such as diet are thought to be linked to CRC initiation and progression 2, 3. Despite growing clinical significance, the detailed molecular mechanisms underlying metastasis in CRC remain poorly understood. The major cause of death in CRC is metastasis. Therefore, it is very important to identify the critical genes involved in the progression of CRC in order to diagnose the disease at an earlier stage using an appropriate biomarker.

We previously reported that FABP5 was highly expressed and involved in metastasis in prostate cancer cells 4, 5, 6, 7, 8, 9. In addition, other studies reported that FABP5 was upregulated in oral squamous cell carcinoma 10, intrahepatic cholangiocarcinoma 11, pancreatic 12, bladder 13, and triple negative breast cancer 14, 15. Moreover, recent proteomic analyses indicated that FABP5 was also upregulated in hepatocellular carcinoma 16 and CRC cells 17. Thus, as *FABP5* is upregulated in several cancer types, it is expected to be a promising prognostic or therapeutic biomarker candidate in these cancers; however, the precise molecular mechanisms underlying FABP5 upregulation and its oncogenic effects in cancer cells remain unclear, despite the extensive efforts of many research groups attempting to clarify the mechanisms. It would be interesting to assess whether the regulatory mechanisms underlying the upregulation of *FABP5* gene expression and the functions of FABP5 protein in cancer cells are mediated by a common signaling pathway. Further studies on the mechanisms regulating *FABP5* gene expression in cancer cells are now in progress in our laboratory. In particular, although FABP5 is the most upregulated protein in the FABP family consisting of ten isoforms 18, the molecular functions of FABP5 in CRC cells remain poorly characterized. As CRC is a common cancer and a major cause of mortality in men and women, it is very important to elucidate these issues. Therefore, the present study attempted to characterize the functions of FABP5 in CRC cells.

Fatty acid‐binding proteins (FABPs) are members of the intracellular lipid‐binding proteins that bind intracellular hydrophobic ligands such as long‐chain fatty acids. FABPs are involved in fatty acid uptake and transport 18, 19. Recent studies also report that FABPs play roles in the regulation of gene expression, cell growth, and differentiation 20, 21. Several FABPs are upregulated in cancer cells; however, the mechanisms that regulate FABP gene expression and function in cancer cells remain poorly characterized. Recent studies demonstrate that metabolic reprogramming is necessary to sustain cancer cell growth and survival. Alteration in fatty acid metabolism is a hallmark of cancer, and several lines of evidence showed that limiting fatty acid availability controls cancer cell proliferation 22, 23. As fatty acids are required for the formation of membrane components, energy sources, and the production of cellular signaling molecules during cancer cell proliferation, FABPs might play an important role in cellular proliferation.

The present study focuses on the physiological functions of FABP5 in CRC cells and assesses the effects of FABP5 expression on CRC cell progression. Results suggest for the first time that high‐level FABP5 promotes cell proliferation and metastatic potential in CRC cells.

## Materials and methods

### Reagents

Oligonucleotides and siRNAs were synthesized commercially at Integrated DNA Technologies (IDT, Coralville, IA, USA). GW0742 and GW1929 were purchased from Sigma‐Aldrich (St. Louis, MO, USA), and GSK‐3787 was from Focus Biomolecules (Plymouth Meeting, PA, USA). The antibody to FABP5 was established as described previously 24. The antibodies to p21^WAF1/Cip1^, p53, phospho‐p53 (Ser15), c‐MYC, AKT, phospho‐AKT (Ser473), and β‐actin were purchased from Cell Signaling Technology (Danvers, MA, USA). The antibody to α‐tubulin was purchased from Santa Cruz Biotechnology (Santa Cruz, CA, USA), and HRP‐conjugated goat anti‐rabbit and anti‐mouse IgG were purchased from Enzo Life Sciences (Farmingdale, NY, USA).

### Cell culture and siRNA transfection

Human CRC cell lines (Caco‐2, DLD‐1, LoVo, and HCT116) were cultured in Dulbecco's modified Eagle's medium (Thermo Scientific, Rockford, IL, USA). Human normal colon fibroblasts (CCD‐18Co) were cultured in Eagle's minimum essential medium (Sigma‐Aldrich). All media were supplemented with 10% fetal bovine serum and antibiotic/antimycotic solution (Nacalai Tesque, Kyoto, Japan), and cells were maintained at 37 °C in an atmosphere of 5% CO_2_. Knockdown of FABP5 gene by siRNA was conducted as follows: cells were transfected with 20 nm negative control siRNA or FABP5 siRNA (IDT, HSC.RNAI.N001444.12.1 and HSC.RNAI.N001444.12.7) using Lipofectamine RNAiMAX (Thermo Scientific) according to manufacturer instructions.

### Quantitative real‐time PCR (Q‐PCR)

Total RNA was extracted using the TRI Reagent (Molecular Research Center, Cincinnati, OH, USA), and cDNAs were synthesized from 1 μg of total RNA using the ReverTra Ace qPCR RT Master Mix (Toyobo, Osaka, Japan). Quantitative real‐time PCR (Q‐PCR) analyses were performed with the StepOne Real‐Time PCR system (Applied Biosystems, Foster City, CA, USA) using THUNDERBIRD SYBR qPCR Mix (Toyobo).

### Western blotting

Cells were lysed in RIPA buffer with protease inhibitor cocktail (Nacalai Tesque). Equivalent amounts of protein were fractionated by SDS/PAGE. Immunoblotting was carried out using the appropriate antibodies. Signals were detected using chemiluminescent substrate (Thermo Scientific) with the Image Quant LAS4000 Mini (GE Healthcare Life Sciences, Pittsburgh, PA, USA).

### Cell proliferation assay

Cells were counted to assess proliferation. HCT116 cells were plated onto six‐well plates at a density of 2 × 10^5^ cells/well and transfected with control or FABP5 siRNA, or treated with GSK‐3787. Cells were counted at 24, 48, and 72 h after transfection or treatment.

### Cell cycle analysis

Cell cycle distribution was assessed by flow cytometry after staining cells with propidium iodide. Briefly, floating and adherent cells were collected, washed with ice‐cold PBS and fixed with 70% ethanol. The cells were then treated with stain solution (100 μg·mL^−1^ RNase A, 1% Triton X‐100, 40 mm sodium citrate, and 50 μg·mL^−1^ propidium iodide) for 1 h at room temperature in the dark. The stained cells were analyzed using a FACSCalibur flow cytometer (BD Biosciences, San Jose, CA, USA).

### Apoptosis assay

The cells transfected with FABP5 or negative control siRNA were collected, diluted in PBS and fixed with BD Cytofix/Cytoperm Fixation and Permeabilization Solution for 20 min on ice in the dark. The cells were then washed with washing buffer and reacted with anti‐active caspase‐3 antibody (BD Biosciences) for 1 h at room temperature. Next, the cells were washed with washing buffer and reacted with FITC‐conjugated anti‐rabbit IgG (Jackson ImmunoResearch, West Grove, PA, USA) for 30 min at room temperature in the dark. After the reaction, the cells were diluted in PBS, and flow cytometry was performed with a FACSCalibur flow cytometer. The data were analyzed using the CellQuest software (BD Biosciences). For each sample, 3 × 10^4^ cells were recorded.

### *In vitro* invasion assay

The *in vitro* invasion assay was performed using BioCoat Matrigel invasion chambers (24‐well plate, 8 μm pore size; BD Biosciences). Briefly, HCT116 cells transfected with FABP5 or negative control siRNA were cultured in DMEM supplemented with 5% FBS for 48 h before the assay. 5 × 10^4^ cells were resuspended in serum‐free DMEM and seeded into the upper chamber, and the lower compartment was filled with DMEM supplemented with 10% FBS. After 24 h, the invasive cells were fixed and stained using the Diff‐Quik Stain Kit (Sysmex, Kobe, Japan). The invasive cells were counted in five random fields per chamber (× 200 magnification). Assays were conducted in duplicates for each experiment, and repeated three times.

### Statistical analysis

Data were expressed as the means ± standard deviation (SD) from at least three independent experiments. Statistical analysis was performed using the Student's *t*‐test. *P* < 0.05 was considered statistically significant.

## Results and discussion

### High expression of FABP5 in CRC cells

**Figure 1:**
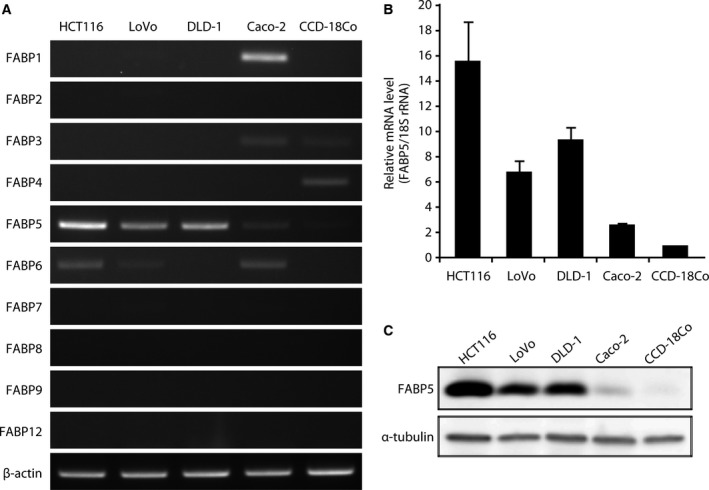
FABP5 is overexpressed in human CRC cells. (A) Semiquantitative analysis of the expression of *FABP* genes by RT‐PCR in human CRC cell lines (HCT116, LoVo, DLD‐1, and Caco‐2) and normal colon fibroblast (CCD‐18Co) cells. β‐actin served as a loading control. The data shown are representative of three independent experiments. (B) Relative expression levels of *FABP5* mRNA in normal and cancer cells were analyzed by quantitative real‐time PCR (Q‐PCR). The results shown are the means ± SD of three independent experiments. (C) Western blot analysis of FABP5 protein levels in HCT116, LoVo, DLD‐1, Caco‐2, and CCD‐18Co cells. Whole cell lysates were prepared and subjected to western blotting. The data shown are representative of three independent experiments.

Although several FABPs are upregulated in cancer cells, the FABP isoforms expressed in CRC cells have not been identified. We first examined the expression of FABP subtypes in normal colon fibroblast cells (CCD‐18Co) and CRC cell lines (HCT116, LoVo, DLD‐1 and Caco‐2). As shown in Fig. 1A, FABP1 (L‐FABP) and FABP3 (H‐FABP) were expressed in Caco‐2 cells. FABP6 (IBABP) was expressed in all cell lines at lower levels, which is consistent with recent studies that demonstrated that FABP6 was upregulated in CRC 25, as was a longer FABP6 variant (IBABP‐L) with 49 additional NH_2_‐terminal amino acid residues 26. The functional differences between IBABP and IBABP‐L in CRC cells remain unclear. Further studies on this issue are now in progress in our laboratory. Interestingly, one of the FABP isoforms, FABP5, is predominantly expressed in all CRC cell lines tested, especially in malignant CRC lines (HCT116, LoVo, and DLD‐1), suggesting that FABP5 might play a critical role in the malignancy of CRC. Next we measured the levels of FABP5 mRNA and protein in CCD‐18Co cells and CRC cell lines. As shown in Fig. 1B,C, FABP5 is markedly upregulated in CRC cells, especially in the highly metastatic HCT116 cells, compared to the nonmetastatic Caco‐2 cells 27, 28 and normal colon fibroblasts (CCD‐18Co). These results clearly suggest that the *FABP5* gene is upregulated in CRC cells and that its level of expression may correlate positively with disease stage.

### Effect of FABP5 expression on cell proliferation, cell cycle, and apoptosis in CRC

**Figure 2:**
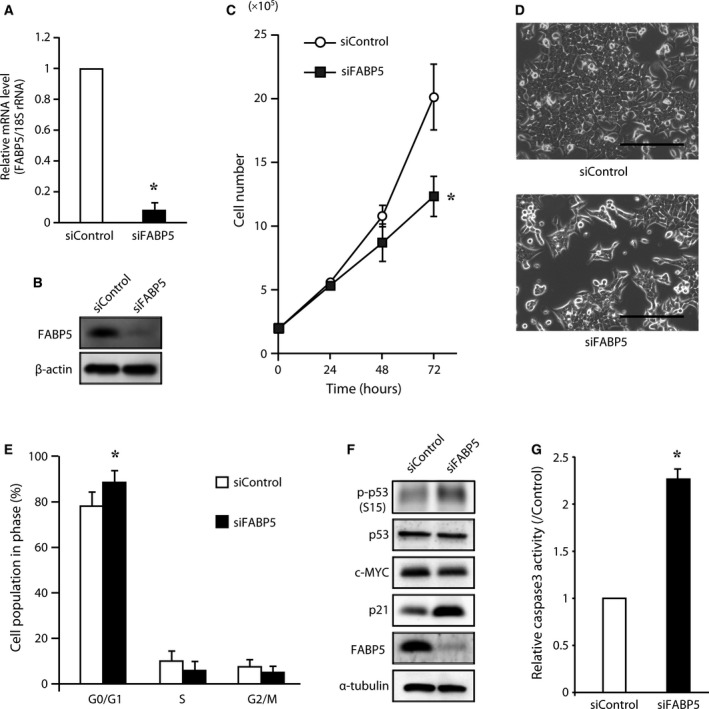
FABP5 knockdown decreases CRC cell growth. HCT116 cells were transfected with siRNA against FABP5. Two different siRNA oligos were used and similar results were obtained. After 72 h, the expression of FABP5 mRNA (A) and protein (B) was evaluated by Q‐PCR and western blotting, respectively. (C) Cell proliferation assay. HCT116 cells were seeded on 6‐well culture plates and transfected with FABP5 siRNA or negative control siRNA. Cells were counted at denoted time points after transfection. The data are expressed as the means ± SD of three independent experiments. (D) Representative images of cells transfected with negative siRNA (upper panel) and FABP5 siRNA (lower panel) 72 h after transfection. Scale bar, 200 μm (E) Cell cycle analysis after FABP5 knockdown. Cell cycle distribution was analyzed. The phase fraction (%) is shown in the graph. (F) FABP5 siRNA increased p21 level. Lysates from HCT116 cells were analyzed by western blotting using specific antibodies to FABP5, p21, c‐MYC, p53, and phospho‐p53 (Ser15). α‐tubulin was used as a loading control. The western blot data shown are representative of three independent experiments. (G) Assay for caspase‐3 activity after transfection with FABP5 siRNA or negative control siRNA was performed. The values represent the rate of induction of apoptosis compared to control (siControl). *, significantly different from siControl, *P* < 0.05.

Although the expression of FABP5 in CRC cells has been reported 17, the molecular mechanisms underlying the function of FABP5 in CRC cell progression are unknown. Thus, to evaluate the possible roles of FABP5 in CRC cells, we first examined the effect of FABP5 on cell proliferation. HCT116 cells were transfected with FABP5‐specific or control siRNA, and a cell proliferation assay was performed. We confirmed that FABP5 expression was significantly reduced at both the mRNA and protein levels 72 h post‐transfection (Fig. 2A,B). Knockdown of FABP5 expression significantly suppressed cell proliferation (Fig. 2C,D). Similar results in LoVo cells were obtained (data not shown). To further investigate the physiological functions of FABP5 in cell cycle progression, we conducted a cell cycle analysis and an apoptosis assay. Cell cycle analysis revealed that knockdown of FABP5 expression resulted in a marked increase in G1/G0 phase population (Fig. 2E). To clarify the functions of FABP5 in cell cycle progression, we assessed FABP5 knockdown‐induced p53 Ser15 phosphorylation and p21^WAF1/Cip1^ upregulation, a key regulator of cell cycle arrest 29. As expected, phospho‐p53 (Ser15) and p21^WAF1/Cip1^ protein were dramatically elevated (about threefold) in HCT116 cells transfected with FABP5 siRNA compared to cells transfected with negative control siRNA (Fig. 2F). We also found that the transcription factor c‐MYC, a negative regulator of p21^WAF1/Cip1^ 30, was downregulated by FABP5 knockdown (Fig. 2F). To assess whether knockdown of FABP5 expression induces apoptotic cell death, a cell apoptosis assay based on the evaluation of caspase‐3 activity by flow cytometry was performed. As illustrated in Fig. 2G, knockdown of FABP5 expression significantly activated caspase‐3, up to 2.2 times compared to control. Cell growth suppression upon knockdown of FABP5 can be attributed to p21‐mediated G1 arrest and apoptosis. These results indicate that FABP5 might play a critical role in cell proliferation, and this is the first report showing a relationship between overexpression of FABP5 and CRC progression.

### Effect of FABP5 expression on cell invasion in CRC

**Figure 3:**
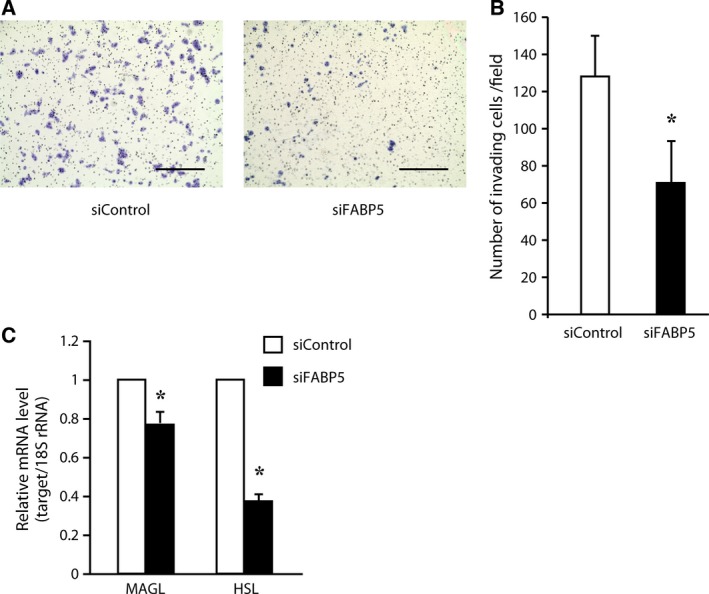
FABP5 knockdown decreases the invasive potential. FABP5 knockdown decreases the invasion of HCT116 colon cancer cells. Cells were induced to invade through Matrigel‐coated membranes. The invasive cells were fixed, stained and counted. Representative images of three independent experiments are shown in (A). Scale bar, 400 μm. The numbers of invasive cells are shown in (B). The data shown are the means ± SD of three independent experiments. (C) HCT116 cells were transfected with siRNA against FABP5 or negative control siRNA. Exactly 72 h after transfection, *MAGL* and *HSL* mRNA levels were determined by quantitative real‐time PCR (Q‐PCR). *, significantly different from siControl, *P* < 0.05.

We examined the effect of FABP5 expression on invasion in CRC cells using an *in vitro* invasion assay. As shown in Fig. 3A,B, knockdown of FABP5 significantly decreased the number of cells invading through the Matrigel‐coated membrane. This finding strongly suggests that FABP5 might regulate the invasiveness of CRC cells during metastasis. Although FABP5 promotes invasion in cancer cells, the molecular mechanism remains unknown. Cancer cells change lipid metabolism to lipogenesis, which promotes malignancy 31. Triacylglycerides are stored in lipid droplets and are used to generate energy or act as second messengers in cancer cells. A recent report showed that monoacylglycerol lipase (MAGL) plays a critical role in liberating stored fats to promote cancer progression 32. In addition, this enzyme produces free fatty acids, leading to the induction of specific lipids such as prostaglandin E2 (PGE2) and lysophosphatidic acid (LPA), which act as signaling molecules in the migration and invasion of cancer cells. Interestingly, this enzyme is highly expressed in aggressive cancer cells and regulates a fatty acid network that promotes migration, invasion, and survival 32. As FABP5 is involved in binding and sorting free fatty acids, as well as in transporting them to the appropriate compartments in the cell, it would be interesting to examine whether FABP5 functionally associates with MAGL‐dependent signaling pathway components to promote invasion in CRC cells. Furthermore, as shown in Fig. 3C, knockdown of FABP5 expression significantly suppressed MAGL and hormone‐sensitive lipase (HSL) expression levels. Importantly, HSL, a rate‐limiting enzyme in diacylglycerol (DG) hydrolysis, generates the MAGL substrate monoacylglycerol. This finding suggests that FABP5 might be involved in MAGL‐dependent signaling in CRC cells. A recent study reported that the overexpression of FABP5 in oral squamous cell carcinoma increased cell invasiveness by increasing the expression of matrix metalloprotease‐9 (MMP‐9) 10; however, knockdown of FABP5 expression did not significantly suppress MMP‐9 gene expression in the present experimental conditions (data not shown), suggesting that FABP5 may not be involved in the invasiveness mediated by MMP‐9.

### The functional association of FABP5 with PPAR β/δ signaling might not be a major growth‐promoting pathway in CRC cells

**Figure 4:**
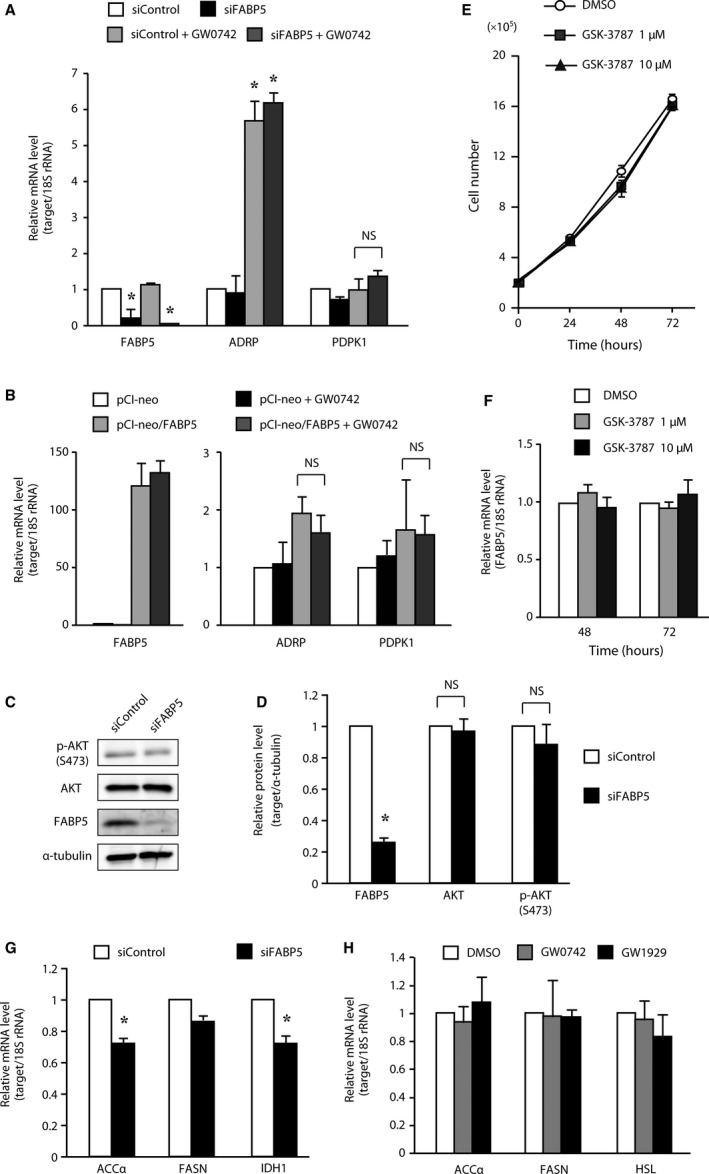
High‐level FABP5 promotes CRC cell growth via functional association with a novel signaling pathway other than PPARδ signaling. (A) HCT116 cells were transfected with siRNA against FABP5 or negative control siRNA. Exactly 48 h after transfection, cells were treated with GW0742 (1 μm, 24 h). *FABP5, ADRP, PDPK1* mRNA levels were determined by quantitative real‐time PCR (Q‐PCR). (B) Caco‐2 cells were transfected with pCI‐neo empty vector or pCI‐neo/FABP5 expression vector. Exactly 48 h after transfection, cells were treated with GW0742 (1 μm, 24 h). *FABP5, ADRP, PDPK1* mRNA levels were determined by Q‐PCR. (C) Western blot analyses using specific antibodies to FABP5, AKT, and phospho‐AKT (Ser473). α‐tubulin was used as a loading control. The western blot data shown are representative of three independent experiments. (D) The intensity of FABP5, AKT, and p‐AKT(S473), quantified by densitometric analysis of three independent experiments, were represented as fold‐change relative to control siRNA, normalized to α‐tubulin. (E, F) HCT116 cells were treated with GSK‐3787 (1 or 10 μm). Cells were counted (E) and the expression of *FABP5* mRNA was evaluated by Q‐PCR (F) at denoted time points after treatment. (G) HCT116 cells were transfected with siRNA against FABP5 or negative control siRNA. Exactly 72 h after transfection, *ACC*α*, FASN*, and *IDH1* mRNA levels were determined by Q‐PCR. (H) HCT116 cells were treated with GW0742 or GW1929 (1 μm, 24 h). *ACC*α*, FASN*, and *HSL* mRNA levels were determined by Q‐PCR. The results shown are the means ± SD of three independent experiments. NS stands for not statistically significant. *, significantly different from non‐treated siControl (A), (B) or siControl (D), (G), *P* < 0.05.

Tumor progression caused by FABP5 overexpression may be the result of the activation of the PPAR β/δ signaling pathway 33, 34, 35. Briefly, FABP5‐PPAR β/δ signaling induced the expression of genes involved in cell growth and survival; however, it is unclear whether FABP5‐dependent cell proliferation in CRC cells is attributable to this mechanism. To assess whether this signaling pathway functions in CRC cells, we examined the effect of FABP5 on PPAR β/δ signaling using GW0742, a synthetic high‐affinity PPAR β/δ agonist. In HCT116 cells, activation of PPAR β/δ with GW0742 resulted in an increase in the mRNA level of adipocyte differentiation‐related protein (ADRP), a well‐characterized PPAR (β/δ and γ) target gene 36, regardless of the FABP5 expression level (Fig. 4A). Furthermore, overexpression of FABP5 and treatment with GW0742 in Caco‐2 cells did not influence ADRP expression level (Fig. 4B). These results suggest that FABP5 does not enhance PPAR β/δ signaling and is not required for PPAR β/δ ligand binding in CRC cells. GW0742 also failed to increase the expression of 3‐phosphoinositide‐dependent protein kinase‐1 (PDPK1), which is a PPAR β/δ target gene 37 and phosphorylates AKT/PKB (protein kinase B), leading to activation of survival signaling (Fig. 4A,B). This result is consistent with recent studies showing no changes in the expression of PDPK1 and phosphorylation of AKT in response to GW0742 in human HaCaT keratinocytes and CRC cells 38, 39, 40. In addition, the levels of phosphorylated AKT were unchanged by knockdown of FABP5 (Fig. 4C,D) and high concentration of GW0742 could not rescue the siFABP5‐mediated cell growth inhibition (data not shown). To further address the question of whether PPAR β/δ mediates the oncogenic activities of FABP5, we investigated whether treatment with PPAR β/δ antagonist affects CRC cell proliferation. Treatment with PPARβ/δ antagonist (GSK‐3787) resulted in no significant decrease in cancer cell proliferation (Fig. 4E) and the expression levels of FABP5 (Fig. 4F). Thus, these results strongly suggest that PPAR β/δ does not mediate the pro‐oncogenic activities of FABP5. Indeed, several lines of evidence have shown that PPAR β/δ signaling does not potentiate the growth of human cancer cell lines and attenuates colon carcinogenesis 39, 40, 41, 42. As shown in Fig. 4A,B,F, the FABP5 expression level did not significantly change in response to GW0742 or GSK‐3787, suggesting that *FABP5* gene expression is upregulated by a PPAR β/δ‐independent signaling pathway in CRC cells. Thus, these data are contradictory to the report showing that FABP5 is involved in PPAR β/δ‐dependent prostate cancer cell growth 34. Moreover, knockdown of FABP5 expression resulted in the downregulation of fatty acid metabolizing genes, such as acetyl‐CoA carboxylase α (ACCα) and isocitrate dehydrogenase 1 (IDH1) (Fig. 4G), suggesting that FABP5 is involved in metabolic alterations in CRC cells. In HCT116 cells, treatment with GW0742 or GW1929 (PPAR γ agonist) had no significant effect on the expression levels of *ACC*α, *FASN*, and *HSL* (Fig. 4H). This result suggests that these lipid‐metabolizing genes were not regulated by PPAR β/δ or PPAR γ in HCT116 cells. As ACCα is a rate‐limiting enzyme in fatty acid biosynthesis, FABP5 might play a critical role in fatty acid metabolism in CRC cells. Importantly, as fatty acids are required for the formation of membrane components, energy sources, and the production of cell signaling molecules during cancer cell proliferation, FABP5 might play a pivotal role in cell proliferation by regulating fatty acid metabolism in CRC cells. Further mechanistic studies of FABP5‐dependent growth in CRC cells are now in progress.

In conclusion, the present study demonstrated that the *FABP5* gene is upregulated in CRC compared to normal colon in a manner that correlates with disease stage. FABP5 significantly promotes cell growth and invasion by a PPAR β/δ‐independent signaling pathway in CRC cells.

## Author contribution

H.F. conceived and supervised the study; K.K. and H.F. designed experiments; K.K., S.S., C.K. and Yu. K. performed experiments; K.K., S.S. and H.F. analyzed data; K.K. and H.F. wrote the paper; Yo. K. provided the samples.

## References

[1] Jemal A , Bray F , Center MM , Ferlay J , Ward E and Forman D (2011) Global cancer statistics. CA Cancer J Clin 61, 69–90. 21296855 10.3322/caac.20107

[2] Markowitz SD and Bertagnolli MM (2009) Molecular origins of cancer: molecular basis of colorectal cancer. N Engl J Med 361, 2449–2460. 20018966 10.1056/NEJMra0804588 PMC2843693

[3] Fearon ER (2011) Molecular genetics of colorectal cancer. Annu Rev Pathol 6, 479–507. 21090969 10.1146/annurev-pathol-011110-130235

[4] Morgan EA , Forootan SS , Adamson J , Foster CS , Fujii H , Igarashi M , Beesley C , Smith PH and Ke Y (2008) Expression of cutaneous fatty acid‐binding protein (C‐FABP) in prostate cancer: potential prognostic marker and target for tumourigenicity‐suppression. Int J Oncol 32, 767–775. 18360704

[5] Adamson J , Morgan EA , Beesley C , Mei Y , Foster CS , Fujii H , Rudland PS , Smith PH and Ke Y (2003) High‐level expression of cutaneous fatty acid‐binding protein in prostatic carcinomas and its effect on tumorigenicity. Oncogene 22, 2739–2749. 12743598 10.1038/sj.onc.1206341

[6] Jing C , Beesley C , Foster CS , Chen H , Rudland PS , West DC , Fujii H , Smith PH and Ke Y (2001) Human cutaneous fatty acid‐binding protein induces metastasis by up‐regulating the expression of vascular endothelial growth factor gene in rat Rama 37 model cells. Cancer Res 61, 4357–4364. 11389060

[7] Jing C , Beesley C , Foster CS , Rudland PS , Fujii H , Ono T , Chen H , Smith PH and Ke Y (2000) Identification of the messenger RNA for human cutaneous fatty acid‐binding protein as a metastasis inducer. Cancer Res 60, 2390–2398. 10811115

[8] Bao Z , Malki MI , Forootan SS , Adamson J , Forootan FS , Chen D , Foster CS , Rudland PS and Ke Y (2013) A novel cutaneous Fatty Acid‐binding protein‐related signaling pathway leading to malignant progression in prostate cancer cells. Genes Cancer 4, 297–314. 24167657 10.1177/1947601913499155 PMC3807643

[9] Forootan FS , Forootan SS , Malki MI , Chen D , Li G , Lin K , Rudland PS , Foster CS and Ke Y (2014) The expression of C‐FABP and PPARγ and their prognostic significance in prostate cancer. Int J Oncol 44, 265–275. 24189640 10.3892/ijo.2013.2166

[10] Fang LY , Wong TY , Chiang WF and Chen YL (2010) Fatty‐acid‐binding protein 5 promotes cell proliferation and invasion in oral squamous cell carcinoma. J Oral Pathol Med 39, 342–348. 20040021 10.1111/j.1600-0714.2009.00836.x

[11] Jeong CY , Hah YS , Cho BI , Lee SM , Joo YT , Jung EJ , Jeong SH , Lee YJ , Choi SK , Ha WS *et al* (2012) Fatty acid‐binding protein 5 promotes cell proliferation and invasion in human intrahepatic cholangiocarcinoma. Oncol Rep 28, 1283–1292. 22825302 10.3892/or.2012.1922

[12] Sinha P , Hütter G , Köttgen E , Dietel M , Schadendorf D and Lage H (1999) Increased expression of epidermal fatty acid binding protein, cofilin, and 14‐3‐3‐sigma (stratifin) detected by two‐dimensional gel electrophoresis, mass spectrometry and microsequencing of drug‐resistant human adenocarcinoma of the pancreas. Electrophoresis 20, 2952–2960. 10546833 10.1002/(SICI)1522-2683(19991001)20:14<2952::AID-ELPS2952>3.0.CO;2-H

[13] Chen R , Feng C and Xu Y (2011) Cyclin‐dependent kinase‐associated protein Cks2 is associated with bladder cancer progression. J Int Med Res 39, 533–540. 21672358 10.1177/147323001103900222

[14] Liu RZ , Graham K , Glubrecht DD , Germain DR , Mackey JR and Godbout R (2011) Association of FABP5 expression with poor survival in triple‐negative breast cancer: implication for retinoic acid therapy. Am J Pathol 178, 997–1008. 21356353 10.1016/j.ajpath.2010.11.075 PMC3070589

[15] Powell CA , Nasser MW , Zhao H , Wochna JC , Zhang X , Shapiro C , Shilo K and Ganju RK (2015) Fatty acid binding protein 5 promotes metastatic potential of triple negative breast cancer cells through enhancing epidermal growth factor receptor stability. Oncotarget 6, 6373–6385. 25779666 10.18632/oncotarget.3442 PMC4467443

[16] Fujii K , Kondo T , Yokoo H , Yamada T , Iwatsuki K and Hirohashi S (2005) Proteomic study of human hepatocellular carcinoma using two‐dimensional difference gel electrophoresis with saturation cysteine dye. Proteomics 5, 1411–1422. 15751005 10.1002/pmic.200401004

[17] Koshiyama A , Ichibangase T and Imai K (2013) Comprehensive fluorogenic derivatization‐liquid chromatography/tandem mass spectrometry proteomic analysis of colorectal cancer cell to identify biomarker candidate. Biomed Chromatogr 27, 440–450. 22991145 10.1002/bmc.2811

[18] Smathers RL and Petersen DR (2011) The human fatty acid‐binding protein family: evolutionary divergences and functions. Hum Genomics 5, 170–191. 21504868 10.1186/1479-7364-5-3-170 PMC3500171

[19] Zimmerman AW and Veerkamp JH (2002) New insights into the structure and function of fatty acid‐binding proteins. Cell Mol Life Sci 59, 1096–1116. 12222958 10.1007/s00018-002-8490-y PMC11337517

[20] Storch J and Corsico B (2008) The emerging functions and mechanisms of mammalian fatty acid‐binding proteins. Annu Rev Nutr 28, 73–95. 18435590 10.1146/annurev.nutr.27.061406.093710

[21] Storch J and Thumser AE (2010) Tissue‐specific functions in the fatty acid‐binding protein family. J Biol Chem 285, 32679–32683. 20716527 10.1074/jbc.R110.135210 PMC2963392

[22] Currie E , Schulze A , Zechner R , Walther TC and Farese RV (2013) Cellular fatty acid metabolism and cancer. Cell Metab 18, 153–161. 23791484 10.1016/j.cmet.2013.05.017 PMC3742569

[23] Santos CR and Schulze A (2012) Lipid metabolism in cancer. FEBS J 279, 2610–2623. 22621751 10.1111/j.1742-4658.2012.08644.x

[24] Watanabe R , Fujii H , Yamamoto A , Hashimoto T , Kameda K , Ito M and Ono T (1997) Immunohistochemical distribution of cutaneous fatty acid‐binding protein in human skin. J Dermatol Sci 16, 17–22. 9438903 10.1016/s0923-1811(97)00615-4

[25] Ohmachi T , Inoue H , Mimori K , Tanaka F , Sasaki A , Kanda T , Fujii H , Yanaga K and Mori M (2006) Fatty acid binding protein 6 is overexpressed in colorectal cancer. Clin Cancer Res 12, 5090–5095. 16951225 10.1158/1078-0432.CCR-05-2045

[26] Fang C , Dean J and Smith JW (2007) A novel variant of ileal bile acid binding protein is up‐regulated through nuclear factor‐kappaB activation in colorectal adenocarcinoma. Cancer Res 67, 9039–9046. 17909007 10.1158/0008-5472.CAN-06-3690

[27] Zirvi KA , Najjar TA and Slomiany BL (1993) Sensitivity of human colon tumor metastases to anticancer drugs in athymic (nude) mice. Cancer Lett 72, 39–44. 8402572 10.1016/0304-3835(93)90008-w

[28] Hamada K , Monnai M , Kawai K , Nishime C , Kito C , Miyazaki N , Ohnishi Y , Nakamura M and Suemizu H (2008) Liver metastasis models of colon cancer for evaluation of drug efficacy using NOD/Shi‐scid IL2Rgammanull (NOG) mice. Int J Oncol 32, 153–159. 18097554

[29] Sherr CJ and Roberts JM (1999) CDK inhibitors: positive and negative regulators of G1‐phase progression. Genes Dev 13, 1501–1512. 10385618 10.1101/gad.13.12.1501

[30] Seoane J , Le HV and Massagué J (2002) Myc suppression of the p21(Cip1) Cdk inhibitor influences the outcome of the p53 response to DNA damage. Nature 419, 729–734. 12384701 10.1038/nature01119

[31] Schulze A and Harris AL (2012) How cancer metabolism is tuned for proliferation and vulnerable to disruption. Nature 491, 364–373. 23151579 10.1038/nature11706

[32] Nomura DK , Long JZ , Niessen S , Hoover HS , Ng SW and Cravatt BF (2010) Monoacylglycerol lipase regulates a fatty acid network that promotes cancer pathogenesis. Cell 140, 49–61. 20079333 10.1016/j.cell.2009.11.027 PMC2885975

[33] Kannan‐Thulasiraman P , Seachrist DD , Mahabeleshwar GH , Jain MK and Noy N (2010) Fatty acid‐binding protein 5 and PPARbeta/delta are critical mediators of epidermal growth factor receptor‐induced carcinoma cell growth. J Biol Chem 285, 19106–19115. 20424164 10.1074/jbc.M109.099770 PMC2885189

[34] Morgan E , Kannan‐Thulasiraman P and Noy N (2010) Involvement of fatty acid binding protein 5 and PPARβ/δ in prostate cancer cell growth. PPAR Res 2010, 234629. 20847935 10.1155/2010/234629 PMC2933898

[35] Levi L , Lobo G , Doud MK , von Lintig J , Seachrist D , Tochtrop GP and Noy N (2013) Genetic ablation of the fatty acid‐binding protein FABP5 suppresses HER2‐induced mammary tumorigenesis. Cancer Res 73, 4770–4780. 23722546 10.1158/0008-5472.CAN-13-0384 PMC4082958

[36] Targett‐Adams P , McElwee MJ , Ehrenborg E , Gustafsson MC , Palmer CN and McLauchlan J (2005) A PPAR response element regulates transcription of the gene for human adipose differentiation‐related protein. Biochim Biophys Acta 1728, 95–104. 15777674 10.1016/j.bbaexp.2005.01.017

[37] Di‐Poï N , Tan NS , Michalik L , Wahli W and Desvergne B (2002) Antiapoptotic role of PPARbeta in keratinocytes via transcriptional control of the Akt1 signaling pathway. Mol Cell 10, 721–733. 12419217 10.1016/s1097-2765(02)00646-9

[38] Borland MG , Khozoie C , Albrecht PP , Zhu B , Lee C , Lahoti TS , Gonzalez FJ and Peters JM (2011) Stable over‐expression of PPARβ/δ and PPARγ to examine receptor signaling in human HaCaT keratinocytes. Cell Signal 23, 2039–2050. 21843636 10.1016/j.cellsig.2011.07.020 PMC3184348

[39] Marin HE , Peraza MA , Billin AN , Willson TM , Ward JM , Kennett MJ , Gonzalez FJ and Peters JM (2006) Ligand activation of peroxisome proliferator‐activated receptor beta inhibits colon carcinogenesis. Cancer Res 66, 4394–4401. 16618765 10.1158/0008-5472.CAN-05-4277

[40] Hollingshead HE , Killins RL , Borland MG , Girroir EE , Billin AN , Willson TM , Sharma AK , Amin S , Gonzalez FJ and Peters JM (2007) Peroxisome proliferator‐activated receptor‐β/δ (PPARβ/δ) ligands do not potentiate growth of human cancer cell lines. Carcinogenesis 28, 2641–2649. 17693664 10.1093/carcin/bgm183

[41] Yang L , Zhou J , Ma Q , Wang C , Chen K , Meng W , Yu Y , Zhou Z and Sun X (2013) Knockdown of PPARδ gene promotes the growth of colon cancer and reduces the sensitivity to bevacizumab in nude mice model. PLoS One 8, e60715. 23593291 10.1371/journal.pone.0060715 PMC3620168

[42] Palkar PS , Borland MG , Naruhn S , Ferry CH , Lee C , Sk UH , Sharma AK , Amin S , Murray IA , Anderson CR *et al* (2010) Cellular and pharmacological selectivity of the peroxisome proliferator‐activated receptor‐beta/delta antagonist GSK3787. Mol Pharmacol 78, 419–430. 20516370 10.1124/mol.110.065508 PMC2939490

